# Forskolin treatment enhances muscle regeneration and shows therapeutic potential with limitations in Duchenne muscular dystrophy

**DOI:** 10.1186/s13395-025-00381-7

**Published:** 2025-05-07

**Authors:** Andreea Iuliana Cojocaru, Kaouthar Kefi, Jean-Daniel Masson, Laurent Tiret, Frederic Relaix, Valentina Taglietti

**Affiliations:** 1 Univ Paris-Est Créteil, INSERM, U955 IMRB, F-94010 Créteil, France; 2 École Nationale Vétérinaire d’Alfort, U955 IMRB, F-94700 Maisons-Alfort, France; 3 EFS, U955 IMRB, F-94010 Créteil, France; 4 AP-HP, Hopital Mondor, Service d’ histologie, 94010 Creteil, France

**Keywords:** Duchenne muscular dystrophy, Muscle stem cells, Forskolin, Muscle regeneration, Fibrosis, Cellular senescence

## Abstract

**Background:**

Duchenne Muscular Dystrophy (DMD) is a progressive neuromuscular disorder characterized by impaired muscle repair. Forskolin (FSK), an adenylyl cyclase activator, has shown potential in enhancing muscle regeneration and limiting muscle stem cell senescence. This study aimed to evaluate the effects of FSK on muscle repair, fibrosis, inflammation, and long-term muscle function in DMD using a preclinical rat model.

**Methods:**

BaCl_2_-induced muscle injury was performed on 6-month-old DMD (R-DMDdel52) and wild-type (WT) rats. FSK was supplied via short-term and long-term administration. Muscle tissues were harvested 14 days post-injury for histological analysis, including hematoxylin and eosin and Sirius red staining. Immunofluorescence was used to assess fibroadipogenic progenitors (FAPs), regeneration, muscle stem cells, and macrophage phenotypes. Moreover, we performed a study by chronically administering FSK to DMD rats from 1 to 7 months of age, either intraperitoneally (IP) or subcutaneously (SC). Functional assessments included grip strength test, in vivo muscle force measurements, plethysmography and electrocardiograms. Post-sacrifice, *Tibialis anterior,* diaphragm and heart tissues were histologically analyzed, to evaluate muscle architecture, fibrosis, and histopathological indices.

**Results:**

FSK treatment significantly improved muscle histology and reduced fibrosis in both uninjured and injured DMD muscles by decreasing the number of FAPs. Long-term FSK treatment in the acute injury model enhanced muscle regeneration, increased MuSC proliferation, and reduced senescence. FSK also modulated inflammation by reducing pro-inflammatory macrophages and promoting a shift to a restorative phenotype. However, despite these histological improvements, FSK treatment from 1 to 7 months resulted in limited functional benefits and worsened ventricular histology in the heart.

**Conclusions:**

FSK shows promising results in improving muscle regeneration and reducing fibrosis in DMD, but concerns remain regarding its limited chronic functional benefits and potential adverse effects on cardiac tissue. Our results highlight the need for optimized adenylyl cyclase activators for therapeutic use in DMD patients.

**Supplementary Information:**

The online version contains supplementary material available at 10.1186/s13395-025-00381-7.

## Background

Duchenne Muscular Dystrophy (DMD) is a severe neuromuscular disorder caused by mutations in the *Dmd* gene, leading to the lack of expression of the Dystrophin protein [1, 2]. Dystrophin plays a crucial role in maintaining the structural integrity of muscle fibers by connecting the cytoskeleton of muscle fibers to the surrounding extracellular matrix (ECM). The lack of functional Dystrophin renders muscle fibers susceptible to damage during contraction, resulting in repeated cycles of degeneration and regeneration [3]. Over time, this process leads to chronic muscle inflammation, fibrosis, and a gradual replacement of muscle with fat and connective tissue, ultimately leading to loss of muscle function.

A critical aspect of the progressive muscle wasting observed in DMD is the compromised ability of muscle to repair. Under normal conditions, muscle regeneration is mediated by satellite cells, a population of muscle stem cells (MuSCs) that resides in a quiescent state until activated by injury. Upon activation, these cells proliferate, differentiate, and fuse to form new muscle fibers or repair damaged ones [4]. However, in DMD, the regenerative capacity of MuSCs is progressively impaired due to increased oxidative stress, chronic inflammation, and repeated cycles of muscle damage [5–7]. The combination of extrinsic and intrinsic factors in dystrophic MuSCs resulted in unbalanced symmetric divisions [8], abnormal mitotic spindle organization [9] and cellular senescence [9–12], characterized by a state of growth arrest and inability to repair muscle tissue effectively. 

Forskolin (FSK), an adenylyl cyclase activator, has been shown to stimulate thyroid-stimulating hormone receptor (TSHR) signaling, which contributes to reducing MuSC senescence. This activation improves the proliferation and function of skeletal muscle stem cells, leading to improved muscle repair and enhanced functional performance in a DMD rat model [11]. While this prior work provides a proof of principle for FSK ability in enhancing DMD muscle repair, the study was limited to short-term treatment. This leaves open critical questions regarding the benefits of FSK chronic treatment and the potential for adverse systemic effects associated with extended use.

This study aims to investigate the effects of FSK administration in a DMD rat model during both acute muscle injury and chronic treatment. By evaluating the impact of FSK on muscle regeneration and on typical DMD hallmarks, as fibrosis and inflammation, this study seeks to provide a comprehensive analysis of the therapeutic potential of FSK. This includes assessing its ability to improve muscle function and slow disease progression in DMD over an extended period. Importantly, this study represents a crucial step toward understanding the long-term benefits and potential limitations of FSK treatment, including the necessary optimization of dosing regimens to avoid adverse effects, which is essential for advancing FSK as a viable therapeutic strategy for DMD to the clinic.

## Methods

### Study design

This study was designed to evaluate the therapeutic potential of Forskolin (FSK) in DMD, using a well-established DMD rat model (R-DMDdel52, [13]) to assess the impact of FSK during acute muscle injury. FSK was administrated for 4 or 12 days after injury by IP injection at 25 mg/kg. This study was conducted under the ethics approval number #35,557–2,022,022,314,489,931. Moreover, we performed a study to examine FSK effects on DMD rats from 1 to 7 months of age, with comprehensive functional assessments such as grip strength, in vivo muscle force measurements, plethysmography, and electrocardiograms, followed by post-sacrifice histological analysis of the *Tibialis Anterior* (TA) muscle, diaphragm, and heart tissues. This approach allowed us to systematically explore FSK effects on muscle architecture and function, with a particular emphasis on its role in modulating fibrosis, inflammation, and stem cell dynamics in the context of DMD. In the chronic study, FSK was administered in two sequential phases. The second phase was designed to recapitulate the previously established minimally effective dose that elicited beneficial effects of FSK, while the first phase aimed to demonstrate the safety and tolerability of FSK at a supra-therapeutic dose. Specifically, rats received FSK at 25 mg/kg for an initial 12-week period. This was followed by an 8-week washout phase designed to assess potential delayed effects of FSK. Finally, FSK was re-administered at the minimally effective dose of 2.5 mg/kg for an additional 4 weeks.

### Rats housing and muscle harvesting

Genotyping was performed on DNA extracted from tail or ear biopsies. The PCR mix included Dream Taq 10X buffer, dNTPs, forward primer (5′-CTAACGCATTTAAAATATGCTGTCA-3′), reverse primer (5′-GTTGGCTTAGCTCAACAACCAAGAT-3′), DreamTaq polymerase, and approximately 150 ng of DNA. PCR was conducted using a Thermal Cycler 2720 with the following protocol: initial denaturation at 95 °C for 5 min, followed by 40 cycles of 95 °C for 10 s, 60 °C for 30 s, and 72 °C for 45 s, with a final extension at 72 °C for 5 min. Deletion of exon 52 was detected on a 2% agarose gel. The rats, all male and including both wild type and DMD, were housed in a pathogen-free facility with 12-h light/dark cycles with food and water provided ad libitum in compliance with European Directive 2010/63/EU. At the appropriate time points, rats were euthanized via CO₂ asphyxiation, and muscles were immediately harvested. The TA, diaphragm, and heart tissues were excised, and frozen in isopentane cooled in liquid nitrogen. Cryosections were cut at a thickness of 7 µm using a cryostat Leica CM1850 and stored at −80 °C until further analysis.

### Hematoxylin & eosin and sirius red

Cryosections were subjected to H&E by rinsing and staining them with hematoxylin for 5–10 min, which is followed by rinses in tap water and bluing in saturated lithium carbonate solution for a few seconds to remove excess stain. After another rinse, the sections are stained with eosin for 1–3 min. Finally, the stained sections are dehydrated through graded alcohols, cleared in xylene, and coverslipped with a permanent mounting medium.

Sirius red staining was conducted by dehydration in 90% ethanol for 5 min and incubation in picrosirius red solution for 30 min, followed by differentiation in acidified water. Stained sections were dehydrated, cleared in xylene, and mounted with a permanent mounting medium. Sirius Red Fast Green was performed by fixing in 4% paraformaldehyde (PFA; Carlo-Erba, 415,691) solution for 30 min, followed by 4 min of incubation in a Sirius Red Fast Green solution made with Direct Red 80 (Sigma-Aldrich, 365,548), Fast Green FCF (Sigma-Aldrich, F7252) dissolved in Picric Acid Solution (Sigma-Aldrich, P6744). Sections were then quickly rinsed in acidified water, dehydrated progressively in ethanol, fixed in xylene and mounted with a permanent mounting medium. Images were captured using the light microscope Axio Imager, and the extent of fibrosis was quantified by measuring the area of Sirius red-positive regions using ImageJ. The histopathology was determined by superimposing onto the image of the entire muscle section a grid of 10,000 µm^2^. At each grid intercept (spaced every 100 µm), the underlying tissue is manually evaluated and categorized based on predefined histological features, such as inflammation, necrotic fibers, or normal muscle fibers. The percentage of intercepts corresponding to pathological features is calculated relative to the total number of intercepts overlying healthy tissue, resulting in the histopathology index. This index represents the proportion of muscle tissue affected by pathological changes.

### FAP isolation and cell culture

TA were isolated from 6-month-old WT and DMD rats and digested for 2 h at 37 °C in HEPES-buffered saline (HBSS; pH 7.3) with dispase II (3 U/ml; Roche, 4,942,078,001) and collagenase A (0.5 U/ml; Roche, 10,103,586,001). The cell suspension was filtered through a 70 µm strainer, centrifuged at 600 g for 5 min at 4 °C, and resuspended in DMEM/F-12 (GlutaMAX; ThermoFisher, Gibco, 31,331,028) supplemented with 20% fetal bovine serum (FBS; ThermoFisher, Gibco, A5256701, Lot 2,575,617) and penicillin/streptomycin (100 mg/ml). Cells were plated for 3 h, and non-adherent cells were cultured in DMEM/F-12 with 20% FBS for 5–7 days. After 5–7 days, cells were incubated with CD45-PE-Cy7 (BD Pharmingen, 561,588), CD31-PE-Cy7 (ThermoFisher, Invitrogen, 50–112–3278), CD29-Pacific Blue (BioLegend, 102,224), and α7-integrin-FITC (CliniSciences, AM20011FC-N) for 30 min at room temperature. Labeled cells were washed with HBSS (pH 7.3), and FAPs (CD45⁻CD31⁻α7⁻CD29⁺) were sorted using a FACSAria (BD Pharmingen). FAPs were plated at 3,000 cells/cm^2^ density and cultured in 20% FBS in DMEM/F-12 for 24 h. Cells were then treated with dimethyl-sulfoxide vehicle (DMSO, Sigma-Aldrich, D8418) or 5, 20 and 50 μM FSK (Sigma-Aldrich, F3917), with medium refreshed every 3 days. Proliferation was assessed on day 7 using a 2-h pulse of 10 µM EdU (Click-iT PLUS Kit, Life Technologies, C10640).

### Immunofluorescence of cultured FAPs

Cells were fixed with 4% PFA for 10 min at 4 °C and then permeabilized with 0.5% Triton X-100 for 10 min. After blocking with 5% bovine serum albumin (BSA), cells were incubated overnight at 4 °C with Collagen I-III (Abcam, ab34710) and αSMA (R&D Systems, #IC1420P). After washing with phosphate-buffered saline (PBS), cells were incubated with appropriate secondary antibodies for 45 min at room temperature and nuclei were counterstained with DAPI (Sigma-Aldrich, D9542). Fluorescence was analyzed with a LSM800 confocal microscope.

### Macrophages isolation and cell culture

Bone marrow was isolated from the tibias of adult WT and DMD rats (6-month-old) by flushing it into a 50 mL tube with 5 mL of DMEM. The suspension was centrifuged at 200 g for 5 min at 4 °C, and the supernatant was discarded. The cell pellet was resuspended in bone marrow culture medium, composed of 10% FBS in DMEM supplemented with 50 ng/ml of CSF1 (Gibco, PMC2044), and filtered through a 100 µm strainer. Cells were plated at a density of 15,000 cells/cm^2^ and incubated at 37 °C with 5% CO₂. On day 5, cells were treated with vehicle (DMSO, Sigma-Aldrich, D8418) or 20 μM FSK (Sigma-Aldrich, F3917) for 48 h.

### RNA extraction and qPCR

RNA was extracted using the NucleoSpin® RNA kit (Macherey–Nagel) and reverse transcribed using SuperScript™ III Reverse Transcriptase (ThermoFisher, 18,080,093). qPCR was performed on a StepOne™ Real-Time PCR System (Applied Biosystems) using the following primers: Actb For TGTCACCAACTGGGACGATA, Actb Rev GGGGTGTTGAAGGTCTCAAA, TNFα For CAGTTCCATGGCCCAGACC, TNFα Rev TACGACGTGGGCTACGGG, IL10 For: AGTGGAGCAGGTGAAGAATG, IL10 Rev GAGTGTCACGGCTTCTATG. Expression levels were normalized to Actb as a housekeeping gene, with controls serving as the reference for treated cells in both WT and DMD groups.

### Immunostaining of cryosections

Sections were fixed in 4% paraformaldehyde for 10 min, followed by permeabilization with 0.5% Triton X-100. After blocking with 5% bovine serum albumin (BSA) in PBS, sections were incubated overnight at 4 °C with primary antibodies specific for regenerating myofibers (eMHC, MYH3 Antibody sc-53091, Santa Cruz), FAPs (PDGFRα, Tebubio, 250E-AB-22250), MuSCs (Pax7, DSHB), and macrophages (CD68, ab31630, Abcam; CD206, ab64693, Abcam). The following day, sections were washed and incubated with appropriate secondary antibodies conjugated with fluorophores. Nuclei were counterstained with DAPI, and coverslips were mounted using an anti-fade mounting medium. Fluorescent images were captured using LSM800 confocal microscope (Zeiss), and positive cells were quantified using ImageJ.

### Grip strength test

To assess muscle function, grip strength tests were performed on rats using a grip strength meter from Bioseb. Each rat was allowed to grip a metal T-bar with its forelimbs, and the maximum force exerted before releasing the grid was recorded. Five repetitions were conducted for each rat, and the highest value was selected as the representative measurement. This value was then normalized to the cube of the tibial length.

### In vivo* muscle force measurements*

Muscle function was assessed using the Aurora 1300A 3-in-1 Whole Animal Muscle System. Subcutaneous EMG electrodes were employed to stimulate the TA, with careful attention paid to electrode placement and stimulation parameters to ensure optimal results. The primary measurement was the torque force generated by the animal's hind limb, which was normalized against relative muscle mass. Isometric contraction assays were performed by stimulating the muscle at increasing frequencies until a plateau in force generation was observed. The peak force for each frequency was recorded and plotted.

### Electrocardiogram

Cardiac function was assessed using Emka technologies ECG telemetry system, where rats were fitted with a jacket to hold electrodes for continuous monitoring of heart activity. The ECG signals were recorded over 15 min on conscious rats. Data were analyzed using ECGavg software and QTpeak corrected (QTpc) values were calculated as QTpc = QTp/(RR/f)1/2, with f equal to the mean RR interval of each evaluated rat [14].

### Whole-body plethysmography

Whole-body plethysmography was conducted using the Emka technologies system equipped with the IOX2 respiratory analysis software. Rats were placed in a chamber, designed to allow unrestrained movement, where respiratory parameters were measured based on pressure changes induced by the animal's breathing. The system included temperature and humidity transducers to ensure precise data collection. Respiratory function was recorded continuously for a duration of 30 min, capturing parameters such as tidal volume and end expiratory pause. Data were monitored in real-time, providing an assessment of the rat respiratory function.

### Data correction and statistical analysis

To account for inter-individual variability in organ mass due to growth differences, organ weights were normalized to the cube of tibia length (TL^3^), reflecting the volumetric nature of mass. Correction factors were derived from previously established linear regressions between TL^3^ and each variable in wild-type animals. Statistical analyses were performed using GraphPad Prism 10.3.1 software. For comparisons involving injured versus uninjured muscle, a two-way ANOVA was conducted, with post-hoc tests applied as appropriate to identify significant differences. When comparing groups, such as WT, DMD, DMD treated with FSK IP, and DMD treated with FSK SC, a one-way ANOVA was used. Post-hoc Tukey’s multiple comparisons test was performed to identify specific group differences. We used a two-way ANOVA to compare TA, heart, and EDL weights normalized on TL^3^. Results were considered statistically significant if the p-value was less than 0.05 and showed in the graphs. Data are presented as mean ± standard error of the mean (SEM).

## Results

### FSK improved muscle architecture and fibrosis in DMD rats upon acute injury

**Fig. 1:**
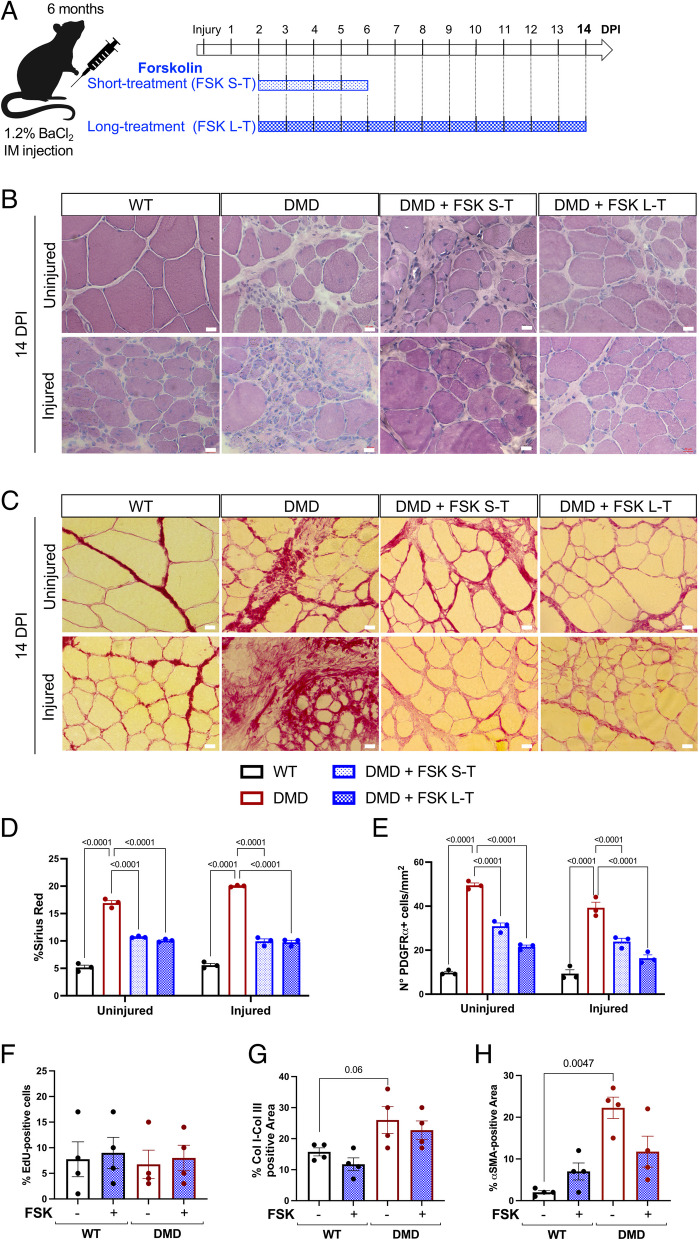
Morphological analysis of uninjured and injured muscles upon FSK treatments. **A** Scheme of FSK administration on DMD rats for a short (4-day) or long (12-day) treatment starting at 2 days post-injury (DPI). FSK was daily administered by IP injection at 25 mg/kg. **B**, **C** H&E (B) and SR (SR) on TA harvested at 14 DPI from WT, DMD, DMD treated with FSK short term (FSK S-T) or long term (FSK L–T). Scale bar 20 µm. **D** Quantification of SR positive area. **E** Quantification of the number of PDGFRa + cells per mm^2^. **F**–**H** Quantifications of FAP proliferation (F), Col I-Col III (G) and αSMA (H) positive area

BaCl_2_-induced muscle injury was performed on 6-month-old R-DMDdel52 and wild-type (WT) rats and muscles were analyzed 14 days post-injury (DPI) to evaluate tissue repair upon acute injury. We sought to determine whether FSK could affect skeletal muscle regeneration upon acute muscle injury. We performed a short-treatment (FSK S-T) for 4 days and a long-treatment (FSK L–T) for 12 days from 2 DPI on DMD rats by intraperitoneal injections (Fig. 1A). Injured and uninjured controlateral TA were harvested from each rat and histologically analyzed. Muscle histology was evaluated by hematoxylin and eosin (H&E) and Sirius red (SR) staining, revealing an improved muscle architecture in DMD rats treated with FSK for both uninjured and injured muscles (Fig. 1B, C). Significant fibrosis was observed in DMD uninjured muscles compared to WT, while it decreased in DMD with both short and long FSK treatment (Fig. 1D). Fibrosis was not significantly increased at 14 DPI in WT and DMD injured muscles, and its build-up remained limited upon FSK administration (Fig. 1D). Given that fibrosis is produced by FAPs marked by PDGFRa expression, we quantified the number of PDGFRa-positive interstitial cells showing a decrease in the presence of FAPs in DMD muscles of FSK-treated rats for short or long duration (Fig. 1E, Supplementary Fig. 1A). Next, we sought to investigate whether FSK had a direct impact on FAP proliferation and fibrogenic commitment. To address this, we isolated FAPs from WT and DMD muscles and treated them with FSK in vitro. We quantified FAP proliferation and their conversion into fibroblasts by analyzing the expression of type I and III Collagen (Col I, Col III) and α-smooth muscle actin (αSMA), which are markers commonly used to identify FAPs that have differentiated into fibroblasts [15]. FSK had no effect on FAP proliferation (Fig. 1F) or fibrotic conversion (Fig. 1G, H, Supplementary Fig. 1B), as the levels of both ColI-III and αSMA remained unchanged with FSK treatment. Also, no differences were observed when examining different concentrations of FSK (Supplementary Fig. 1C-E).

Altogether, the results demonstrated that FSK treatment ameliorated muscle architecture and reduced fibrosis in both uninjured and injured DMD muscles, by decreasing the number of FAPs. However, the lack of a direct effect of FSK on FAP proliferation or fibrotic conversion in vitro suggests that its antifibrotic actions are not direct, potentially mediated through other cellular or molecular mechanisms within the muscle environment.

### FSK enhanced muscle regeneration and reduced senescence in DMD rats

**Fig. 2:**
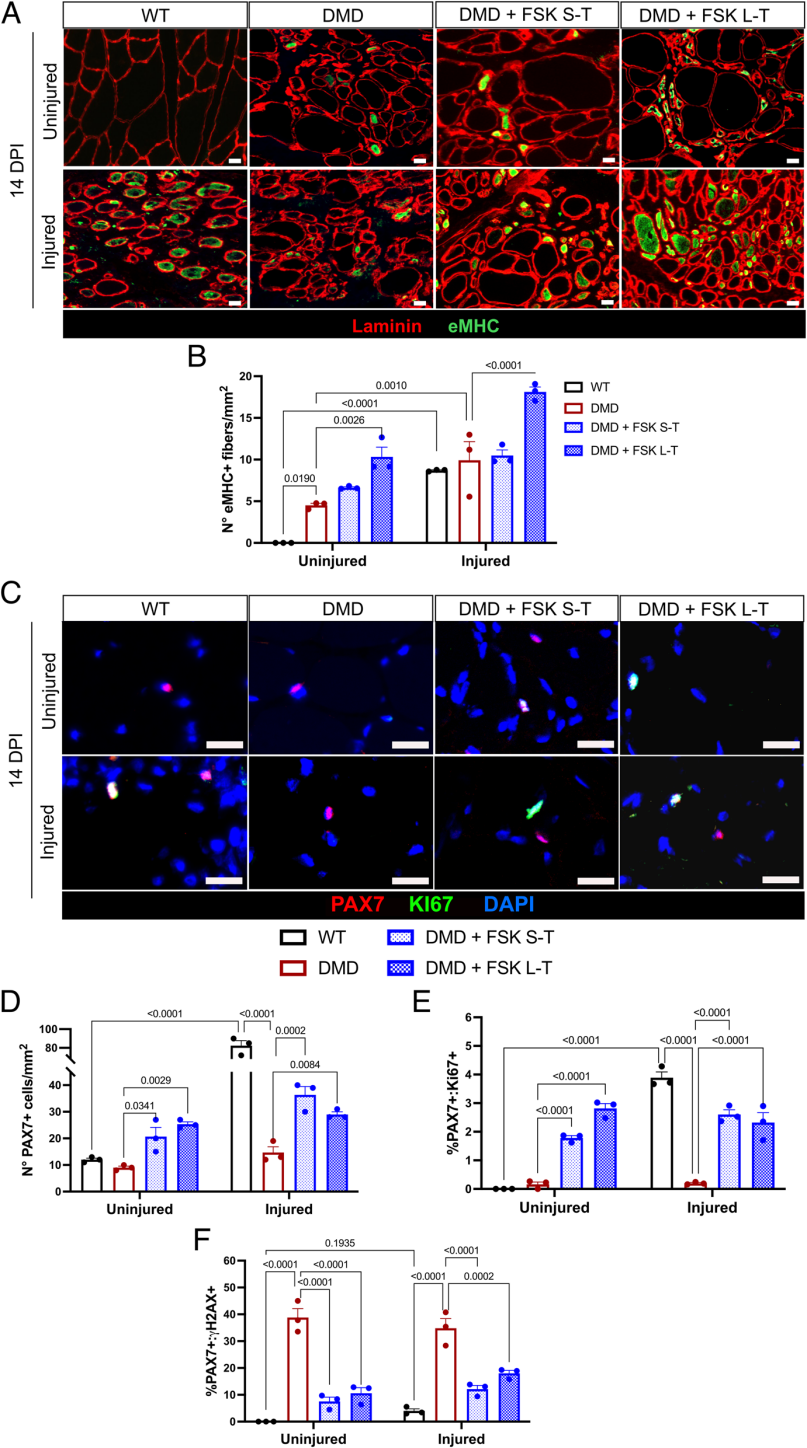
Assessment of muscle regeneration and MuSC senescence in injured and control muscles upon FSK treatments. **A** Immunofluorescence for Laminin (red) and eMHC (green) on TA harvested at 14 DPI from WT, DMD, DMD treated with FSK short term (FSK S-T) or long term (FSK L–T). Scale bar 20 µm. **B** Quantification of the number of eMHC + fibers per mm^2^. **C** Immunofluorescence for KI67 (red), PAX7 (green) and DAPI on TA harvested at 14 DPI from WT, DMD, DMD treated with FSK short term (FSK S-T) or long term (FSK L–T). Scale bar 25 µm. **D** Quantification of the number of PAX7 + cells per mm^2^. **E** Quantification of the percentage of PAX7 + : KI67 + cells. F) Quantification of the percentage of PAX7 + : γH2AX + cells

Next, we tested whether FSK would have an impact on muscle repair by analyzing the number of newly regenerated myofibers marked by the expression of embryonic myosin heavy chain (eMHC, Fig. 2A). As expected, uninjured DMD muscles have a higher number of eMHC-positive fibers compared to WT, reflecting the continuous cycles of degeneration and regeneration typical of this disease (Fig. 2B). Interestingly FSK L–T, but not S-T, enhanced muscle regeneration in uninjured DMD muscles (Fig. 2B). The effect of FSK L–T was even more evident upon acute muscle injury by the increased numbers of eMyHC fibers compared to DMD controls (Fig. 2B). Then we evaluated the number of PAX7 + MuSCs (Fig. 2C). Immunofluorescence staining showed more PAX7 + MuSCs in DMD uninjured muscles treated with FSK for both a short and long period (Fig. 2D). At 14DPI, the number of PAX7 + MuSCs was increased in WT but not in DMD control muscles. Both FSK S-T and L–T treatments significantly enhanced the number of MuSCs in injured muscles compared to DMD controls. Further quantifications of active PAX7 + MuSCs, marked by the expression of KI67, showed that FSK treatments increased the proliferation rate of DMD MuSCs in both uninjured and injured muscles, while their expansion was limited in control injured DMD MuSCs (Fig. 2E). To validate the functional impairment of DMD MuSC proliferation, we assessed the number of senescent PAX7 + MuSCs coexpressing the serine-139 H2AX histone (γH2AX), the second most used marker of cellular senescence after βgal [16]. Control DMD rat muscles, either uninjured or injured, showed the highest number of PAX7 + :γH2AX + MuSCs, indicating cellular senescence. Strikingly, PAX7 + :γH2AX + MuSCs were significantly reduced in TA muscles from FSK treated DMD rats with or without injury (Fig. 2F). To conclude, FSK, especially with long-term treatment, boosted muscle regeneration, increased MuSCs proliferation, and reduced MuSCs senescence in DMD rats.

### FSK modulated inflammation by reducing macrophages and promoting a shift to a restorative phenotype

**Fig. 3:**
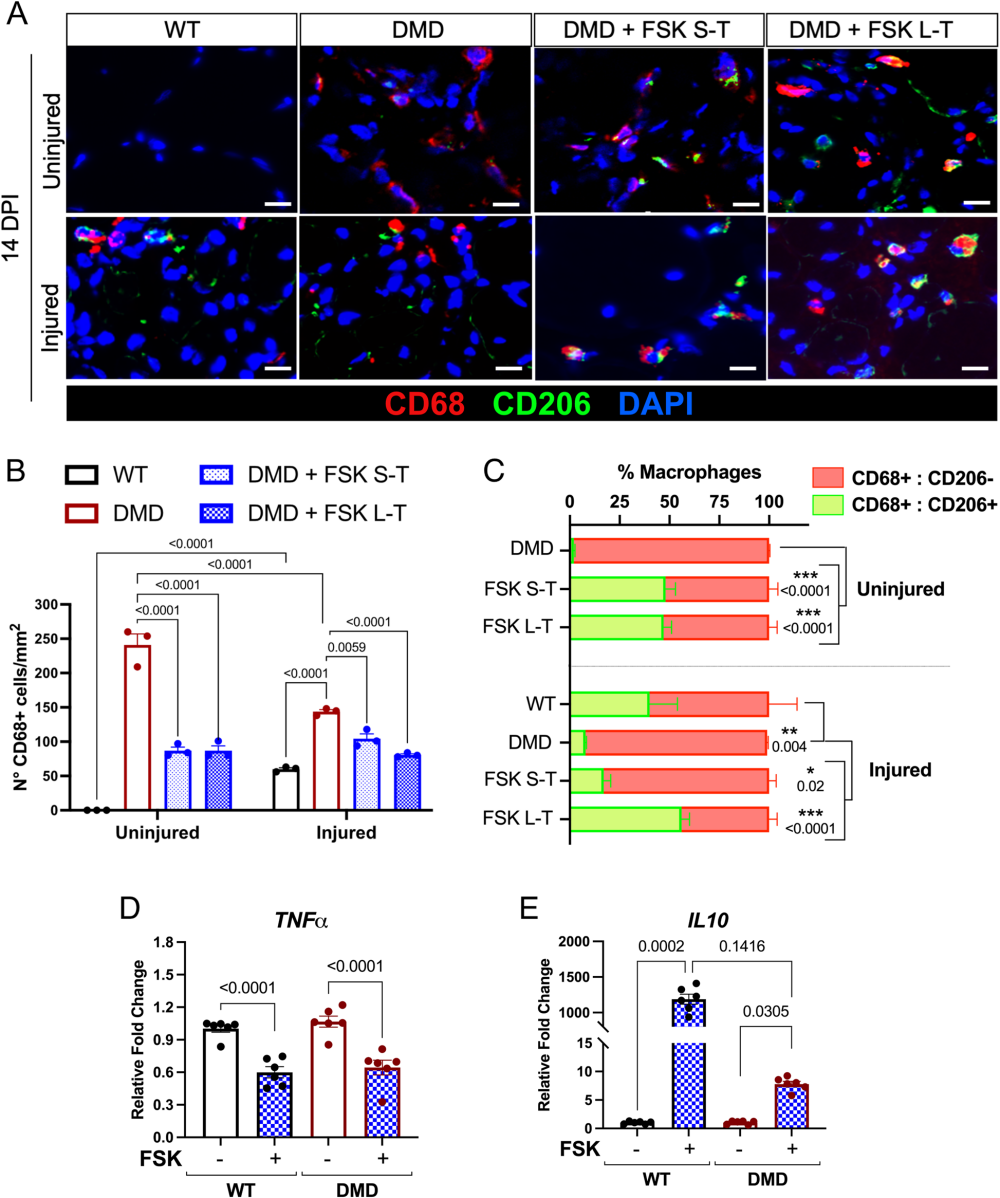
Evaluation of macrophages infiltration in injured and control muscles upon FSK treatment. **A** Immunofluorescence for CD68 (red) and CD206 (green) on TA harvested at 14 DPI from WT, DMD, DMD treated with FSK short term (FSK S-T) or long term (FSK L–T). Scale bar 50 µm. **B** Quantification of the number of CD68 + cells per mm^2^. **C** Quantification of the percentage of CD68 + : CD206- and CD68 + : CD206 + cells. **D**, **E** qPCR analysis of *Tnfα* (D) and *Il10* (E) on BMDM from both WT and DMD rats upon treatment with FSK

To assess the impact of FSK treatment on inflammation, we performed immunofluorescence staining for the pan-macrophage marker CD68 (Fig. 3A). The number of CD68 + macrophages was significantly higher in DMD uninjured muscles compared to WT muscles, consistent with the inflammation characteristic of the disease. FSK short and long treatment could decrease the total number of macrophages in DMD uninjured muscles. Surprisingly, injured TA muscles in DMD rats exhibited fewer CD68 + macrophages compared to their uninjured counterparts, while the number of macrophages was increased in injured WT muscles compared to their controlateral muscle without injury (Fig. 3B). FSK treatment led to a lower number of CD68 + macrophages in both uninjured and injured muscles of DMD rats (Fig. 3B). We next addressed the switch between inflammatory (M1) and restorative (M2) macrophage subpopulations, marked as CD68 + :CD206- (M1) and CD68 + :CD206 + (M2) respectively [17]. M1 macrophages are pro-inflammatory and contribute to the early phases of muscle injury by clearing debris and amplifying the immune response, while M2 macrophages are anti-inflammatory and play a key role in muscle repair by promoting tissue regeneration and resolution of inflammation. Prior to injury, the majority of macrophages in DMD muscle were of inflammatory phenotype (CD68 + :CD206-), as previously shown [13]. Short and long FSK treatments increased the proportion of restorative M2 macrophages (Fig. 3C). 14 days after injury, DMD muscles contained a lower percentage of CD68 + :CD206 + macrophages compared to WT and this percentage was increased upon short and long FSK administration (Fig. 3C). Next, we isolated bone marrow derived macrophages (BMDM) from both WT and DMD rats at 6 months of age and cultured them with or without FSK. After treatment, we evaluated the expression level of the pro-inflammatory (*Tnfα*) and anti-inflammatory (*Il10*) cytokines. FSK treatment reduced the elevated expression of *Tnfα* and increased *Il10* expression in macrophages from both WT and DMD (Fig. 3D-E). Thus, FSK treatment reduced the number of pro-inflammatory macrophages and promotes a shift towards a restorative macrophage phenotype both in vivo and in vitro.

### Chronic FSK treatment showed limited functional improvements

**Fig. 4:**
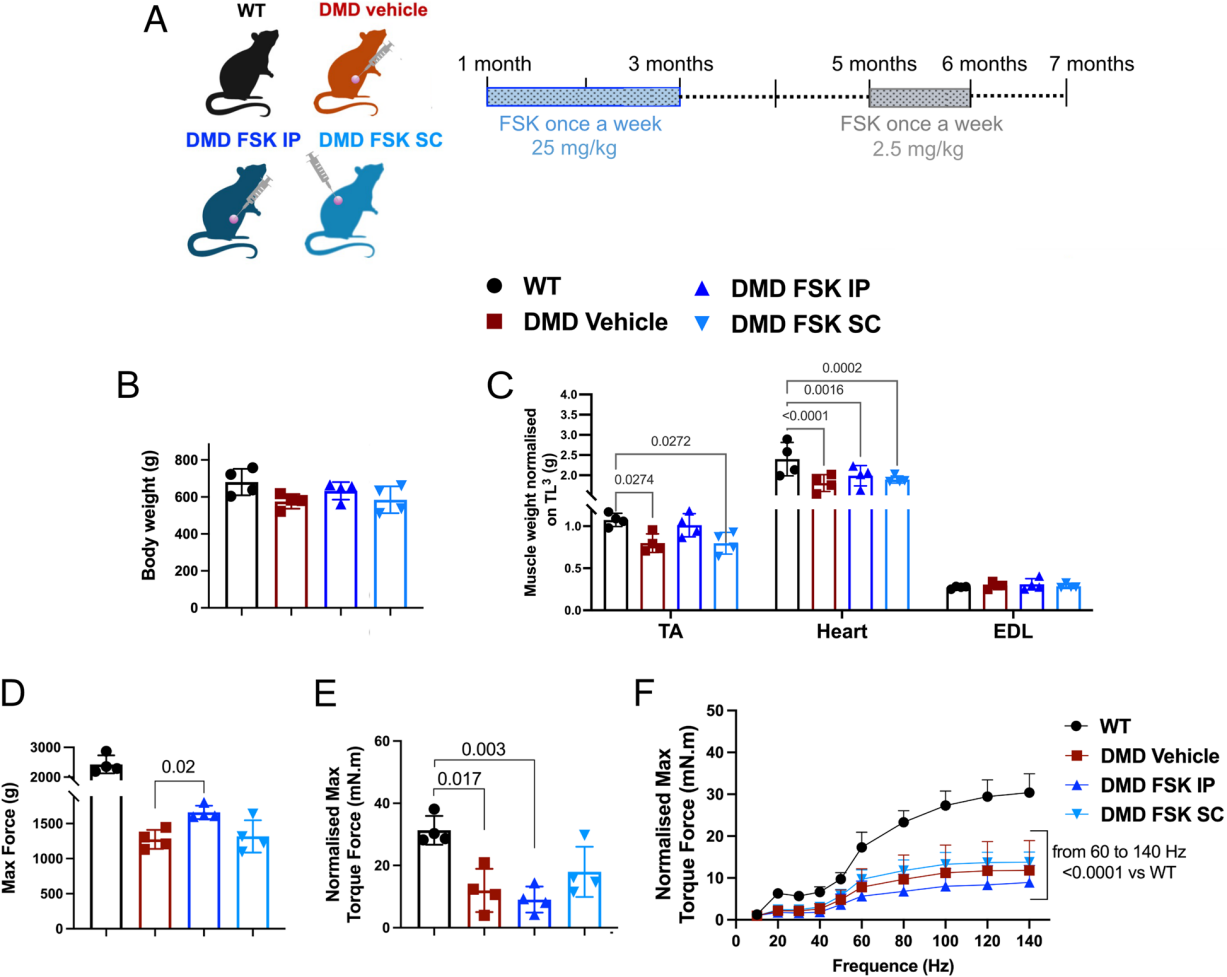
Physiological and functional outcomes following chronic FSK treatment. **A** Scheme of rat injection plan. Forskolin (FSK) was weekly administrated for two months (25 mg/kg) followed by 2 months of wash-out up to 5 months of age. The rats were then injected for another month with FSK (2,5 mg/kg) up to 6 months and then sacrificed at 7 months. FSK was delivered by intraperitoneal (IP) or subcutaneous (SC) injection. **B** Body weight. **C** TA, heart and EDL weights normalized on the cubic tibial length at the end of the treatment. **D** Maximal force normalized on the cubic tibial length by grip test. **E** Normalized Maximal torque force. F) Normalized Maximal torque force as function of muscle stimulation frequency

We next performed a chronic FSK treatment in DMD rats from 1 to 7 months of age, to assess its long-term therapeutic effects during the period when significant muscle atrophy, body weight loss, and functional decline become critical in DMD rats (Fig. 4A). FSK was weekly administrated either IP or SC for two months (25 mg/kg) followed by 2 months of wash-out up to 5 months of age. The rats were then injected for another month with FSK (2,5 mg/kg) up to 6 months and then sacrificed at 7 months. We decided to compare intraperitoneal FSK delivery (IP), as previously described [11] with subcutaneous treatment (SC) to test if we could use the SC route for future experiments. SC administration is preferred for chronic treatments spanning several months, as it is less stressful and painful for the animals compared to IP administration. This approach is important not only from an ethical perspective, but also because stress can potentially influence the study’s outcome. Chronic IP injections could also lead to abdominal adhesions and internal organ damage in the animals. IP-treated DMD rats with vehicle-only were used as controls together with WT littermates. Given our previous observation that FSK treatment had no discernible effect on muscle in WT rats [11], and in consideration of ethical guidelines, FSK-treated WT animals were not included in the present study. After 6 months of treatment, no mortality was observed, and there were no significant differences in body weight between the FSK-treated groups (both IP and SC) and vehicle treated DMD rats (Fig. 4B). This indicates that the treatment did not affect rat growth and showed no apparent toxicity. TA and heart mass were reduced in DMD rats and remained unaffected by FSK treatment, regardless of the administration route (Fig. 4C). No changes were observed in EDL weight (Fig. 4C). Before sacrifice, a functional follow-up was conducted for each rat. Only DMD rats treated IP with FSK exhibited a slight but significant increase in grip force (+ 15%) compared to vehicle-treated DMD rats, with no differences observed between control DMD and SC treated animals (Fig. 4D). Using muscle function assessment techniques with Aurora Scientific's system, we observed no difference in maximal force or fatigue between vehicle- and FSK-treated DMD rats (Fig. 4E, F). Torque forces were normalized to muscle weight, revealing no differences between vehicle- and FSK-treated groups, suggesting that the improved grip function is primarily due to increased muscle mass. In summary, IP FSK treatment increased muscle mass and grip strength but resulted in limited functional improvements regarding muscle force and fatigue.

### FSK ameliorated muscle histopathology and reduces fibrosis in DMD rats

**Fig. 5:**
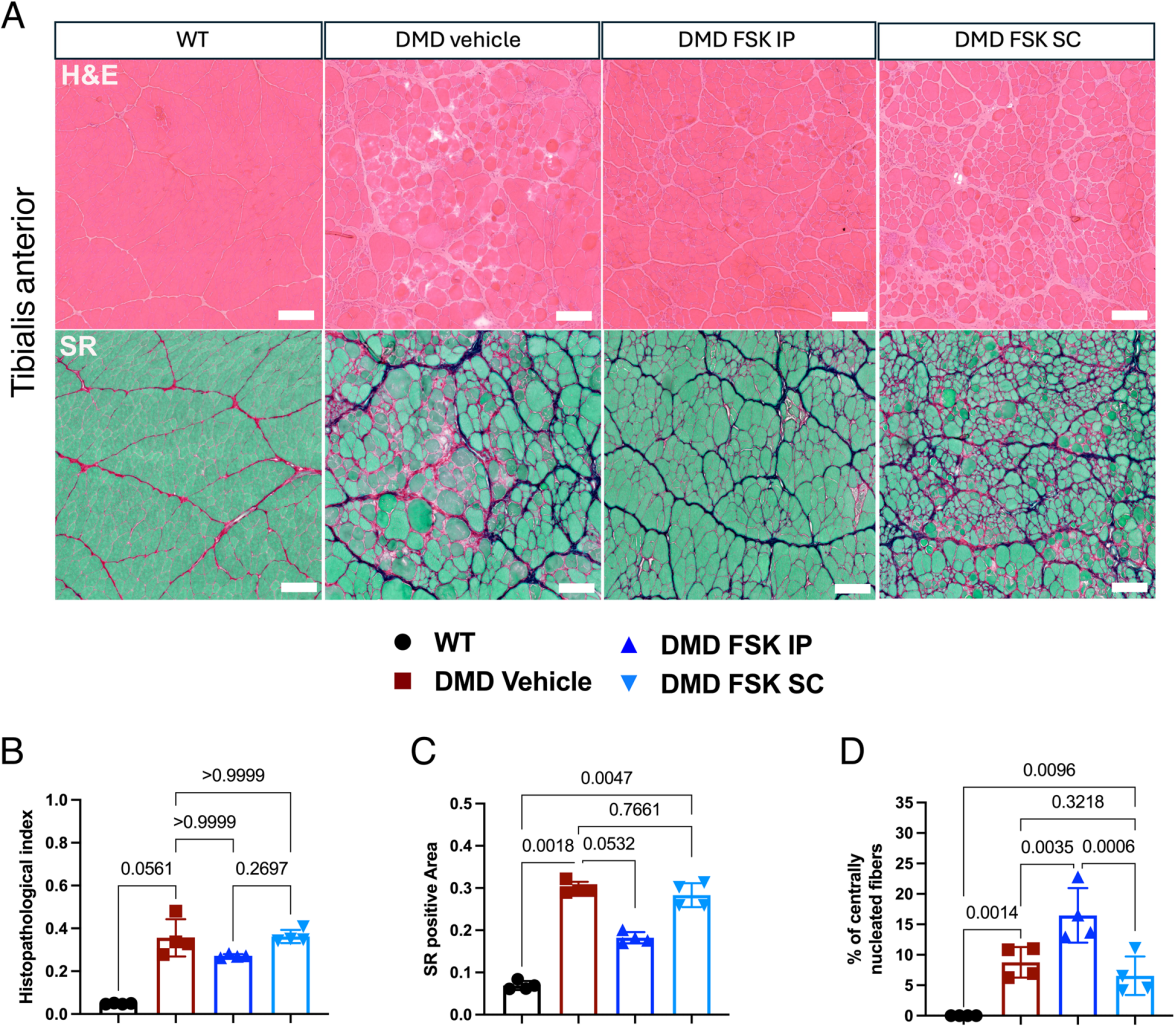
Histopathological evaluation of TA muscles. **A** H&E (upper panels) and SR (lower panels) on TA from WT, DMD vehicle and DMD treated with FSK by IP or SC administration. Scale bar 50 μm. **B** Quantification of the histopathological index. **C** Quantification of fibrosis and SR positive area. **D** Percentage of centrally nucleated fibers

After sacrifice, we harvested the TA muscle and performed H&E and Sirius Red Fast Green staining. Figure 5A shows an overall macroscopic amelioration of muscle morphology in DMD rats treated IP with FSK, as observed by H&E staining, with improved fiber organization and reduced inflammatory infiltrates compared to vehicle-treated DMD rats. However, this was not reflected in a significant improvement of the histopathological index (Fig. 5B), while we observed a reduction in fibrotic deposition in DMD rats treated IP with FSK, as quantified by the Sirius Red-positive area (Fig. 5C). Subcutaneous FSK treatment, contrarily to IP, did not ameliorate the muscle morphology as exhibited by H&E and SR staining. Moreover, there was neither improvement in the histopathological index nor a reduction in the SR positive area. In addition, we quantified the percentage of centrally nucleated myofibers as marker of fibers that have been regenerated over a longer period, compared to eMHC, which is a transient marker for newly regenerated fibers and is gradually replaced by adult MHC isoforms as the fibers mature. The proportion of centrally nucleated fibers was increased in DMD rats treated by intraperitoneal administration of FSK, but not by the subcutaneous route (Fig. 5D). Overall, intraperitoneal FSK treatment improved the dystrophic phenotype in TA muscles, with reduced fibrosis and enhanced muscle histology in DMD rats.

### FSK treatment had no significant impact on diaphragm and respiratory function

**Fig. 6:**
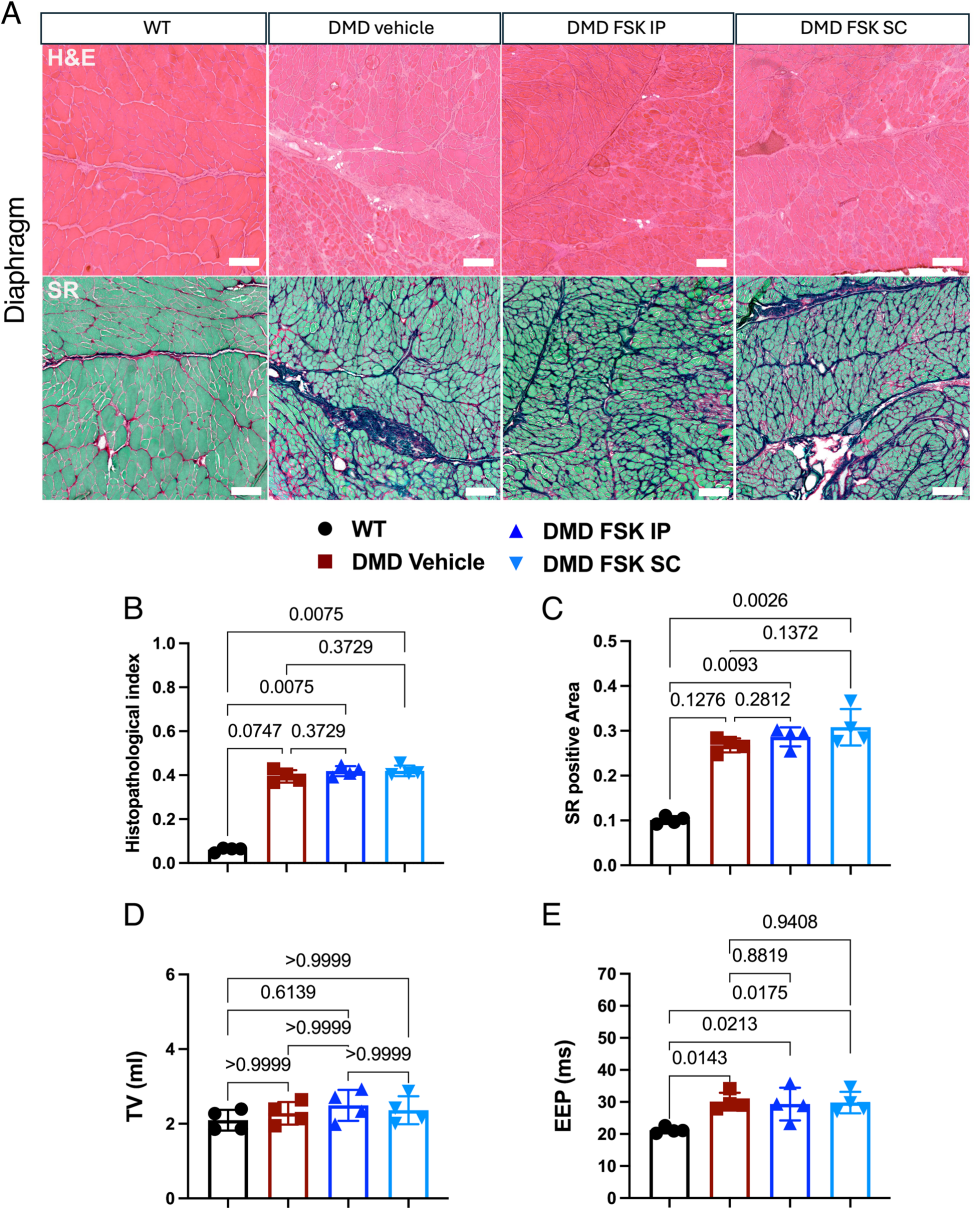
Histopathological evaluation of Diaphragms. **A** H&E (upper panels) and SR (lower panels) on diaphragms from WT, DMD vehicle and DMD treated with FSK by IP or SC administration. Scale bar 50 μm. **B** Quantification of the histopathological index. **C** Quantification of fibrosis and SR positive area. **D** Plethysmography analysis of tidal volume (TV, D) and end expiratory pause (EEP, E)

Next, we analyzed the Diaphragm and observed no major histological changes following FSK administration (Fig. 6A). Indeed, histological hallmarks of the disease were similarly represented in DMD samples, regardless of treatment or route of administration, as indicated by the quantifications of the histopathological index (Fig. 6B). Also, no improvement in fibrosis was observed with the treatment (Fig. 6C), indicating that FSK had no effect on the diaphragm. In addition, we performed plethysmography, a non-invasive technique for analyzing respiratory functions. These analyses revealed that tidal volume remained unchanged in these cohorts of rats (Fig. 6D). However, DMD vehicle-treated rats displayed impaired respiratory functions compared to WT controls, as the end expiratory pause (EEP) increased in dystrophic rats. No significant differences in the EEP were observed between FSK- and vehicle- treated DMD rats (Fig. 6E), indicating that FSK hadn’t any beneficial effect od diaphragmatic function.

### FSK treatment showed negative effects on heart histology in DMD rats

**Fig. 7:**
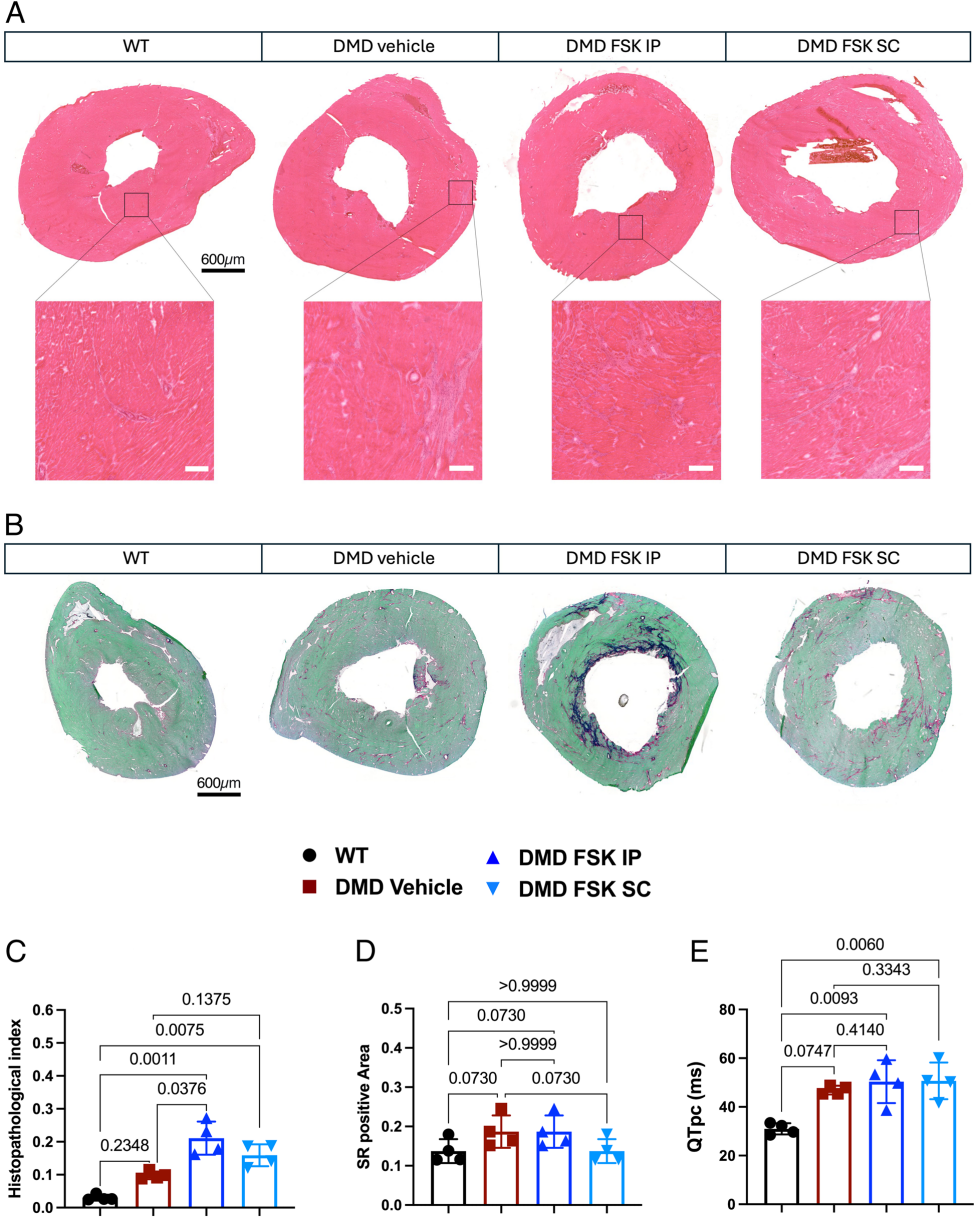
Histopathological evaluation of left and right ventricles. **A** H&E on heart sections from WT, DMD vehicle and DMD treated with FSK by IP or SC administration. Scale bar 600 μm. **B** SR staining on heart sections from WT, DMD vehicle and DMD treated with FSK by IP or SC administration. Scale bar 600 μm. **C** Quantification of the histopathological index. **D** Quantification of fibrosis and SR positive area. **E** ECG evaluation of QTpc value that corresponds to the interval from the Q wave to the peak of the T wave (QTp) corrected with the Bazette’s formula normalized to the average rat RR (QTpc = QTp(eak) / (RR / f)1/2, f = 150 ms)

Finally, we assessed the histology of the heart, focusing on the right and left ventricles (Fig. 7A, B). Overall, no major changes were observed in the histology of the DMD heart treated SC with FSK compared to the vehicle-treated rats, while an increase in the histopathological index was noted for DMD rats treated IP with FSK (Fig. 7C). Next, we evaluated whether the worsening of the heart histopathological index in these rats was due to increased deposition of connective tissue across the sections. Direct quantification of the fibrotic area marked by Sirius Red did not reveal any significant increase in fibrosis in DMD hearts treated IP with FSK, while all the samples presented with a similar Sirius Red positive area regardless of the genotype or treatment (Fig. 7D). Additionally, no differences were observed in the infiltration of CD68 + macrophages within the endocardium (Supplementary Fig. 2A, B), nor in the proportion of resolutive CD68 + :CD206 + macrophages following FSK administration via either the IP or SC route (Supplementary Fig. 2C, D). Finally, we assessed heart function by electrocardiogram, showing that DMD rats developed defects in cardiac conduction as evidenced by an increase in the QTpc interval compared to WT control (Fig. 7E). We found no difference between vehicle- and FSK-treated DMD rats after treatment (Fig. 7E). Altogether, we found that FSK treatment did not improve cardiac function while intraperitoneal FSK administration worsened ventricular histology.

## Discussion

Duchenne muscular dystrophy is characterized by muscle wasting associated with progressive impaired regeneration. Forskolin, an adenylyl cyclase activator, has shown promise in enhancing muscle regeneration, reducing muscle stem cell senescence, and improving muscle performances [11]. Nevertheless, the potential of FSK to enhance regeneration in DMD under conditions of acute injury or during extended FSK treatment, as would be required in actual DMD patient care, was not investigated.

This study aimed to explore the efficacy of FSK in a DMD rat model that exhibits a disease trajectory more comparable to DMD patients than dystrophin-deficient mdx mice [13], with a focus on its effects on muscle regeneration, fibrosis, inflammation, and overall muscle functions. However, we acknowledge that species-specific differences, including pharmacokinetic and metabolic variations, must be considered when interpreting the translational relevance of our findings. We showed that FSK treatment significantly improved muscle architecture and reduced fibrosis in DMD rats following acute injury. Histological analyses revealed enhanced muscle structure and reduced fibrosis in FSK-treated DMD muscles, consistent with improved muscle repair. However, the anti-fibrotic effects of FSK in vivo were not associated with a direct action of FSK on FAP proliferation or fibrotic commitment in vitro, even though previous studies demonstrated that FSK had the potential of limiting the production of ECM proteins and the differentiation into myofibroblasts in various tissues. Specifically, in vitro FSK has been shown to reduce myofibroblast differentiation and ECM secretion in skin and cardiac fibroblasts [18, 19], as well as in 3D models of liver fibrosis [20]. This inhibitory effect of FSK on myofibroblast and collagen synthesis has also been validated in vivo in a carbon tetrachloride-induced liver fibrosis model [21], in a bleomycin-induced pulmonary fibrosis model [22], and in a renal fibrosis model of chronic kidney disease [23]. The inability of FSK to counteract the fibrotic conversion of DMD FAPs in vitro may be attributed to the culture conditions or may suggest that its antifibrotic effects are indirectly mediated, potentially through its impact on other cellular components within the muscle tissue, such as macrophages. Indeed, we observed that FSK reduced the number of M1 pro-inflammatory macrophages by increasing the proportion of M2 anti-inflammatory macrophages, both in vivo and in vitro. In vitro FSK directly influenced macrophage polarization, promoting their shift toward the M2 phenotype. Promoting an anti-inflammatory macrophage phenotype has been shown to be beneficial in the context of DMD, as anti-inflammatory macrophages crosstalk with both FAPs and MuSCs, facilitating tissue repair. Indeed, Juban et al. highlighted that in murine and human DMD muscles, anti-inflammatory macrophages were linked to areas of reduced fibrosis and active myogenesis as they supported MuSC differentiation [24, 25]. In contrast, pro-inflammatory macrophages (Ly6C^pos^) exhibit pro-fibrotic activity in dystrophic muscle, by secreting large amounts of latent TGF-β1 driven by their high expression of LTBP4 [26].

The anti-inflammatory activity of FSK is not exclusive to skeletal muscles. Previous works have shown that FSK reduced inflammation acting on leukocytes, adipocytes, Kupffer cells, and macrophages by inhibiting key inflammatory pathways, such as NFκB and the NLRP3 inflammasome, as well as cytokine production [27–30]. These anti-inflammatory effects align with our in vivo results from the muscle acute injury model, where FSK promoted tissue repair by increasing the number of newly formed myofibers and reducing markers of MuSC senescence in FSK-treated DMD rats. This supports FSK’s role in enhancing muscle regeneration, as previously demonstrated [11].

Despite the promising histological improvements in skeletal muscles within the context of acute injury in DMD, the functional benefits of FSK treatment from 1 to 7 months of age were limited. For this chronic treatment, we employed a two-phase FSK regimen with a high-dose phase (25 mg/kg for 12 weeks) to assess innocuity following a supra-therapeutical dose, followed by an 8-week washout and a re-administration of the minimal effective dose (2.5 mg/kg for 4 weeks) previously shown as beneficial. These dosing choices, delivered both intraperitoneally or subcutaneously, may partly explain the differences between the short-term histological improvements and the more limited long-term functional benefits. Indeed, while IP FSK administration increased TA muscle mass and grip strength in treated DMD rats, it did not significantly improve muscle force or reduce fatigue. Additionally, we observed that IP injections resulted in more effective drug delivery compared to SC injections. This increased efficacy could be attributed to the fact that IP administration allows for faster systemic absorption and broader biodistribution, whereas SC injections may lead to slower and more variable uptake due to local tissue absorption. Our study also revealed that diaphragm and heart did not benefit from FSK treatment, with ventricular histology worsening in DMD rats treated IP with FSK. Given that cardio-respiratory failure is a major cause of morbidity in DMD patients, this finding underscores the challenge of targeting respiratory muscles and heart, which may require alternative or adjunct therapeutic strategies to achieve meaningful benefits. The lack of cardiac benefits is surprising, as FSK has been shown to improve diastolic function in streptozotocin-induced diabetic cardiomyopathy, using a FSK regimen of 2 mg/kg IP for 4 weeks [31], and to alleviate hypertrophic cardiomyopathy with oral doses at 32 and 160 mg/kg for 4 weeks [32]. This discrepancy could be due to the dose and timing of FSK regimen. The limited functional improvements and potential adverse effects on cardiac tissue, along with the lack of efficacy with subcutaneous administration, highlights the need for optimization of the route and dosage of FSK administration, especially for treatments longer then 4 weeks. Furthermore, FSK is a general adenylate cyclase activator and can therefore mimic the activation of various G-protein-coupled receptors (GPCRs) as adrenergic pathways in heart and skeletal muscles [33–38]. In muscle stem cells, FSK can replicate the signaling such as CALCR, Frizzled 7, EP4, GPR116, all of which are crucial for MuSC quiescence, maintenance, or expansion [33, 39–41]. The broad activation of multiple pathways by systemic administration of FSK indicates the need for more targeted and focused approaches in the future. Such approaches should aim to selectively modulate specific adenylate cyclase isoforms within the muscle to enhance therapeutic outcomes while minimizing unintended effects on other tissues and organs.

## Conclusions

Our study demonstrates that FSK exhibited promising effects on enhancing muscle regeneration and reducing fibrosis in a DMD rat model following acute injury. While these findings underscore the therapeutic potential of FSK, they also reveal its limitations in long-term efficacy, particularly regarding muscle function and potential adverse effects on cardiac tissue. Consequently, further research is required in optimizing adenylate cyclase activator administration, dosage, and selectivity to maximize their benefits while minimizing risks, ultimately improving their therapeutic application for DMD treatment.

## Supplementary Information

Supplementary Material 1: Figure 1. Analysis of PDGFRa+ cells upon in vivo and in vitro FSK treatment. A) Immunofluorescence for PDGFRa (red) and Laminin (green) on TA harvested at 14 DPI from WT, DMD, DMD treated with FSK short term (FSK S-T) or long term (FSK L-T). Scale bar 20µm. B) Immunofluorescence for EdU (white), aSMA (red) and type I-III Collagen (green) on sorted FAPs upon treatment with FSK 20 µM. Scale bar 20 µm. C-E) Quantifications of FAP proliferation (C), Col I-Col III (D) and αSMA (E) positive area upon treatment with FSK 5, 20 or 50 µM

Supplementary Material 2: Figure 2. Evaluation of macrophages infiltration in cardiac muscle upon FSK treatment

## Data Availability

No datasets were generated or analysed during the current study.

## Abbreviations

DMD: Duchenne Muscular Dystrophy
FSK: Forskolin
WT: Wild-Type
R-DMDdel52: Rat Model of Duchenne Muscular Dystrophy with a deletion in exon 52
IP: Intraperitoneal
SC: Subcutaneous
TA: Tibialis Anterior
S-T: Short term
L-T: Long term
MuSCs: Muscle Stem Cells
FAPs: Fibro-adipogenic Progenitors
H&E: Hematoxylin and Eosin
SR: Sirius Red
DPI: Days Post-Injury
eMHC: Embryonic Myosin Heavy Chain
ECM: Extracellular Matrix
BMDM: Bone marrow derived macrophages
GPCRs: G-Protein-Coupled Receptors
