# Supplementary figures and images for: Forskolin treatment enhances muscle regeneration and shows therapeutic potential with limitations in Duchenne muscular dystrophy
Source: Skelet Muscle. 2025 May 7;15:12. doi: 10.1186/s13395-025-00381-7 (PMC12057055; doi:10.1186/s13395-025-00381-7)

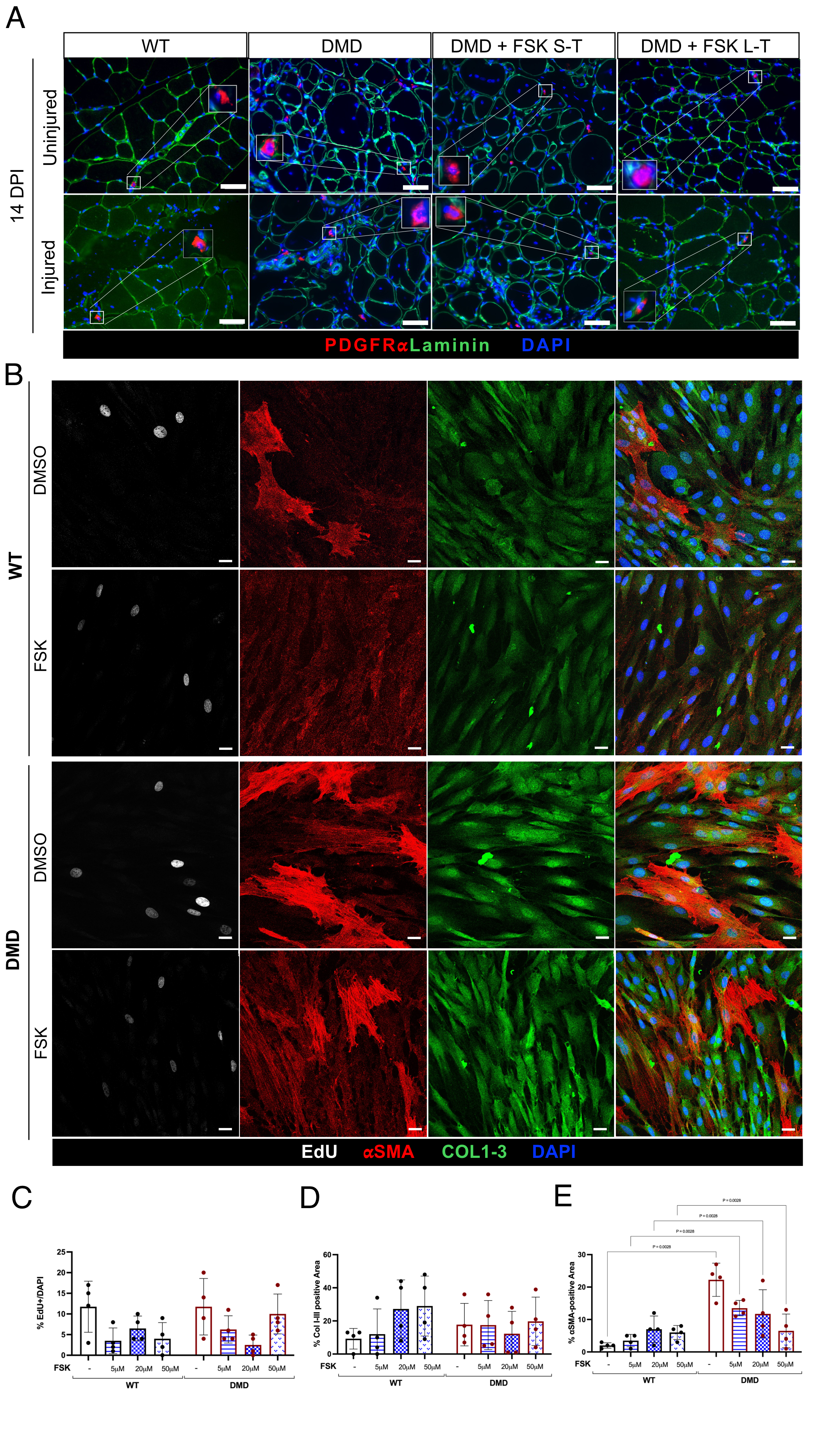

Supplement: Supplementary file 1 — Supplementary Material 1: Figure 1. Analysis of PDGFRa+ cells upon in vivo and in vitro FSK treatment. A) Immunofluorescence for PDGFRa (red) and Laminin (green) on TA harvested at 14 DPI from WT, DMD, DMD treated with FSK short term (FSK S-T) or long term (FSK L-T). Scale bar 20µm. B) Immunofluorescence for EdU (white), aSMA (red) and type I-III Collagen (green) on sorted FAPs upon treatment with FSK 20 µM. Scale bar 20 µm. C-E) Quantifications of FAP proliferation (C), Col I-Col III (D) and αSMA (E) positive area upon treatment with FSK 5, 20 or 50 µM [file 13395_2025_381_MOESM1_ESM.tiff]

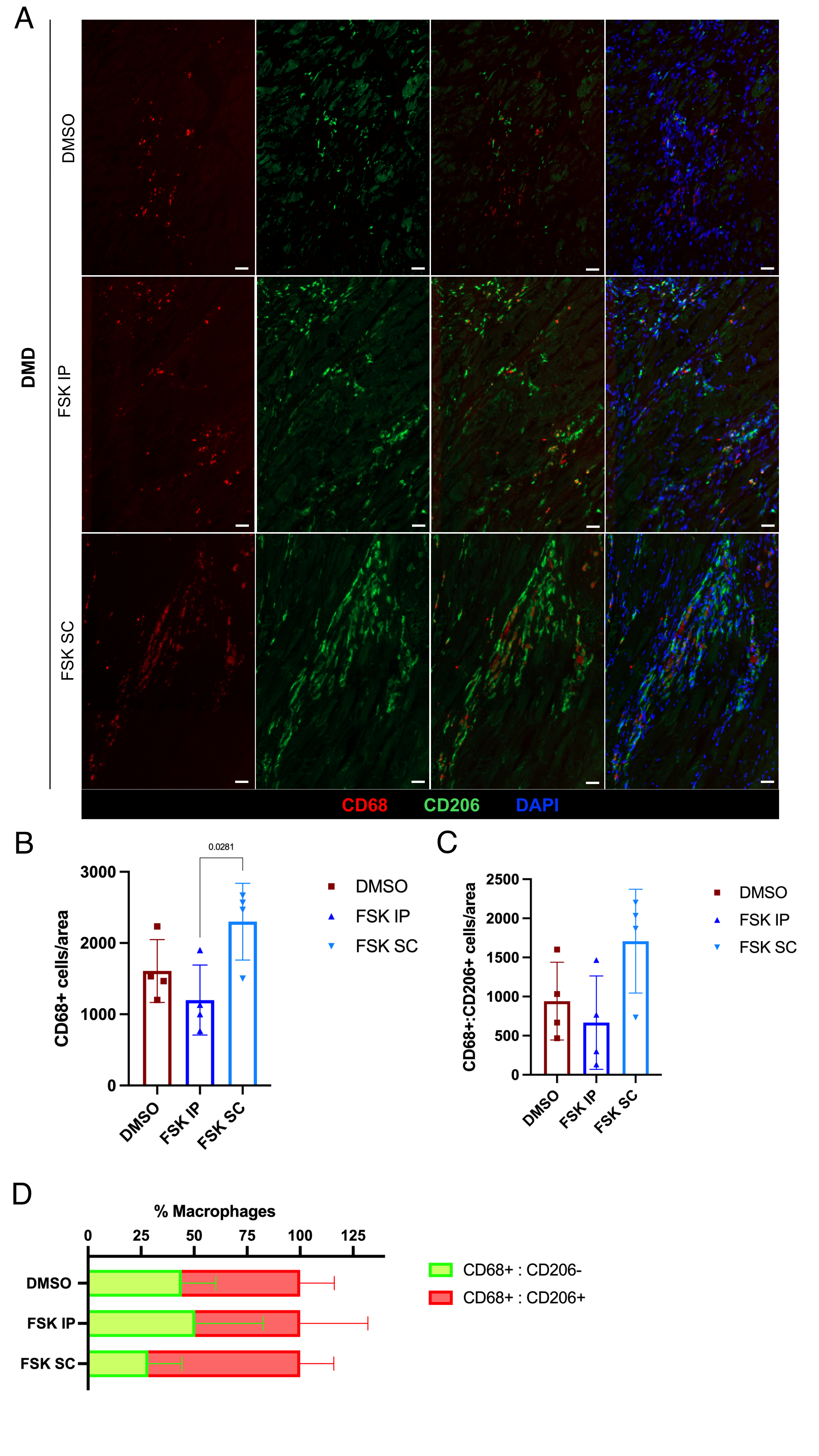

Supplement: Supplementary file 2 — Supplementary Material 2: Figure 2. Evaluation of macrophages infiltration in cardiac muscle upon FSK treatment [file 13395_2025_381_MOESM2_ESM.tiff]
